# Mechanisms Underlying Directional Motion Processing and Form-Motion Integration Assessed with Visual Perceptual Learning

**DOI:** 10.3390/vision6020029

**Published:** 2022-05-31

**Authors:** Rita Donato, Andrea Pavan, Giovanni Cavallin, Lamberto Ballan, Luca Betteto, Massimo Nucci, Gianluca Campana

**Affiliations:** 1 Dipartimento di Psicologia Generale, University of Padova, Via Venezia 8, 35131 Padova, Italy; luca.betteto@studenti.unipd.it (L.B.); massimo.nucci@unipd.it (M.N.); gianluca.campana@unipd.it (G.C.); 2 Human Inspired Technology Research Centre, University of Padova, Via Luzzati 4, 35121 Padova, Italy; lamberto.ballan@unipd.it; 3 Proaction Laboratory, Faculty of Psychology and Educational Sciences, University of Coimbra, Colégio de Jesus, Rua Inácio Duarte 65, 3000-481 Coimbra, Portugal; 4 CINEICC, Faculty of Psychology and Educational Sciences, University of Coimbra, Rua Colégio Novo, 3000-115 Coimbra, Portugal; 5 Dipartimento di Psicologia, University of Bologna, Viale Berti Pichat, 5, 40127 Bologna, Italy; 6 Dipartimento di Matematica, University of Padova, Via Trieste 63, 35121 Padova, Italy; gioca1993@gmail.com

**Keywords:** dynamic Glass patterns, random dot kinematograms, directional motion, non-directional motion, visual perceptual learning, form–motion integration

## Abstract

Dynamic Glass patterns (GPs) are visual stimuli commonly employed to study form–motion interactions. There is brain imaging evidence that non-directional motion induced by dynamic GPs and directional motion induced by random dot kinematograms (RDKs) depend on the activity of the human motion complex (hMT+). However, whether dynamic GPs and RDKs rely on the same processing mechanisms is still up for dispute. The current study uses a visual perceptual learning (VPL) paradigm to try to answer this question. Identical pre- and post-tests were given to two groups of participants, who had to discriminate random/noisy patterns from coherent form (dynamic GPs) and motion (RDKs). Subsequently, one group was trained on dynamic translational GPs, whereas the other group on RDKs. On the one hand, the generalization of learning to the non-trained stimulus would indicate that the same mechanisms are involved in the processing of both dynamic GPs and RDKs. On the other hand, learning specificity would indicate that the two stimuli are likely to be processed by separate mechanisms possibly in the same cortical network. The results showed that VPL is specific to the stimulus trained, suggesting that directional and non-directional motion may depend on different neural mechanisms.

## 1. Introduction

The human and non-human primate visual cortex is divided into two main streams called ventral and dorsal streams [1]. The ventral pathway processes form features, whereas the dorsal stream processes motion features. Ungerleider and Mishkin [1] were the first authors who showed that the visual system is characterized by these two streams that originate at the level of the primary visual cortex (V1) and propagate differently to the parietal area and the inferotemporal area. According to Ungerleider and Mishkin [1], these two visual streams were physically segregated yet functionally independent. However, several later research studies disputed this perspective, advocating an integrated view of the visual brain [2,3,4,5,6,7,8,9].

Dynamic Glass patterns (GPs) [10] and random dot kinematograms (RDKs) are two types of visual stimuli commonly employed to assess the functional properties of the dorsal and ventral streams. A sequence of static GPs is shown in succession to create dynamic GPs. Static GPs are made up of dipoles, which are pairs of dots randomly displayed in a single frame [11]. Each frame in dynamic GPs is characterized by a different arrangement of dipoles. Observers report seeing directional motion when a set of static GPs is displayed fast, even though there is no dipole-to-dipole correspondence between video frames. In addition, the orientation of dipoles can produce many global configurations such as circular, radial, or translational GPs, among others. Dynamic GPs induce a percept of non-directional motion in which the pattern’s global orientation is consistent with the observed apparent directionality. Although dynamic GPs include local and global form aspects determined by dipole orientation, they are primarily connected to motion perception circuits in the brain [12]. Dynamic GPs have been employed in several investigations to offer insight into how form–motion interactions occur in the human visual cortex [2,4,8,9,12,13,14,15,16,17]. RDKs, in contrast to dynamic GPs, are made up of single dots that follow a definite path across the sequence of frames. Fast-moving dots can produce *motion streaks* (also called motion or speed lines) that influence motion perception [3,18,19,20]. Motion streaks are the smeared representation in the visual system of a fast-moving stimulus due to the temporal integration [19]. The human visual system interprets this blurred trail as a form/oriented feature [3]. When the direction of a moving stimulus is uncertain, the visual system can employ motion streaks as a cue to determine a specific motion trajectory [3]. This suggests that the brain cells responsible for processing form cues aid motion perception. The neural substrates of the two forms of motion elicited by dynamic GPs and RDKs have been investigated in neuroimaging and physiological studies, which revealed overlapping cortical brain regions [21,22]. For example, Krekelberg et al. [21] investigated the neural basis of circular and radial dynamic GPs and RDKs using functional magnetic resonance imaging (fMRI). The activation of the human motion complex hMT+ was equivalent for both types of visual stimuli. This suggests that hMT+ does not differentiate between motion induced by form cues in dynamic GPs and motion evoked by motion cues in RDKs. *Cue invariance* is the name given to this occurrence [21]. Donato et al. [13] assessed the causal role of two distinct cortical visual brain regions, V1/V2 and hMT+, in the processing of circular RDKs and circular dynamic GPs. Two groups of participants had to determine whether the coherent dynamic GP or RDK was in the first or second temporal interval (two-interval forced-choice task—2IFC). The sole difference between the two groups was the brain region stimulated: one got online repetitive transcranial magnetic stimulation (rTMS) over V1/V2, while the other group received online rTMS over hMT+. The aim of using rTMS was to temporarily disrupt the functional integrity of the cortical brain region that was activated during the presentation of the visual stimuli. The authors argued that, if V1/V2 and hMT+ played the same functional role in the processing of circular directional and non-directional motion, participants’ performance would have to deteriorate as soon as rTMS was delivered over the brain areas of interest, for both GP and RDK tasks. The authors found, however, that just interfering with the activity of hMT+ had a substantial causal inhibitory effect on discriminating RDKs. Therefore, hMT+ is likely to have a distinct role in the processing of circular dynamic GPs and circular RDKs, but V1/V2 does not have a significant causal function in both classes of visual stimuli.

Psychophysical paradigms that looked at the individuals’ coherence thresholds were also used to study GPs and RDKs [23]. Nankoo et al. [23], for example, investigated how the human visual system processes various combinations of directional motion, non-directional motion, and static stimuli elicited by RDKs, dynamic GPs, and static GPs, respectively. Vertical, horizontal, circular, radial, and spiral configurations were used. The participants were presented a visual pattern that could be an RDK, a dynamic, or a static GP, depending on the experimental session. The task was to determine whether the pattern was coherent or random/noisy (i.e., two-alternative forced-choice task—2AFC). The findings showed that observers perceive dynamic and static GPs more similarly than RDKs, since both GP types had similar detection thresholds when compared to RDKs. Even though neuroimaging research [21] found that dynamic GPs and RDKs have common neural bases, somehow, they appear to be processed differently.

Most of the research into the neural underpinnings of dynamic GPs and RDKs has so far focused on complex configurations like circular and radial patterns. There have been no direct comparisons of the cortical visual regions involved in the processing of simple directional and non-directional motion elicited by dynamic translational GPs and RDKs. To bridge this gap and indirectly shed light on the neural mechanisms underlying dynamic translational GPs and RDKs, we employed visual perceptual learning (VPL). After persistent practice on the same activity, VPL refers to a considerably increased performance in a visual task [24,25,26,27,28,29,30,31]. After a series of training sessions, VPL generates long-lasting perceptual gains [28,29,31,32,33,34,35].

VPL has been used to improve visual functions, such as visual acuity, contrast sensitivity, and it can transfer to other untrained visual stimuli [36,37,38]. Several variables, such as the time interval between training sessions, the task difficulty, the task precision, the number of training sessions, the duration of the training sessions, the participants’ fatigue, and so on, have a significant impact on the type and strength of learning [39,40,41,42,43,44]. Several visual features can be trained including spatial contrast, orientation, motion direction, object recognition, etc. [45,46,47,48,49,50,51,52]. In the current study, VPL was employed to investigate learning transfer between form and motion perception. In particular, the aim was to assess whether VPL on dynamic translational GPs transfers to translational RDKs and vice versa. We trained two groups of participants on the discrimination of dynamic translational GPs and RDKs. We specifically investigated whether an eight-session training develops VPL not only for the learned visual stimuli but also for the non-trained visual stimulus. We predicted that, if the human visual system processes directional and non-directional motion in dynamic translational GPs and RDKs in a similar way and through the same neural circuits, we should be able to generalize VPL to non-trained visual stimuli after a period of training. On the contrary, if there is just a limited overlap in the neural processing of these two classes of visual stimuli, VPL should be either specific to the trained visual stimulus or exhibit partial transfer to the non-trained stimulus.

## 2. Method

### 2.1. Participants

Thirty-one participants took part in the experiment. Specifically, fifteen participants (mean age: 22.3 yrs; SD: 2.74; 11 females and 4 males) took part in the training with RDKs and sixteen (mean age: 24.5 yrs; SD: 3.01; 7 females and 9 males) took part in the training with translational dynamic GPs. All participants were naïve except two of the authors (RD and LB) who participated in the experiment, one in the GPs training group (LB) and the other in the RDKs training group (RD). All participants had normal or corrected to normal vision. In the experiment, viewing was binocular. Participants have been randomly assigned to one of the two groups. Each observer was assigned by chance and in alternation to one of the two different learning groups (i.e., either GPs or RDKs). Participants performed the online experiment using their personal computers. The experiment was run in agreement with the World Medical Association Declaration of Helsinki [53]. The study was approved by the Ethics Committee of the Department of Psychology of the University of Padova (Protocol number: 4112). Written informed consent was obtained before the beginning of the first session.

### 2.2. Stimuli

We used translational dynamic GPs and RDKs. The spatial and temporal characteristics of dynamic GPs were the same as in Pavan et al. [14]. GPs were made of 688 white dots with a width of 0.04 deg on each frame, whereas RDKs were made of 1376 white dots. GPs and RDKs were presented in a circular annulus display (inner radius 0.5°, outer radius 4.5°) on a grey background. Density was 10.95 dipoles/deg^2^ and the length of the dipoles in GPs was 0.18°. Dynamic GPs were made of a set of independent frames where each frame contained a new dipoles’ spatial order, although dipoles’ orientation remained constant [15]. Dynamic GPs induce a perception of directionally ambiguous motion (with alternating opposite directions) that is given by dipoles orientation; however, in dynamic GPs, there is not dipole-to-dipole correspondence between successive frames [15].

RDKs evoked directionally ambiguous motion. The lifetime of the dots was the same as the interval displayed to make a match with dynamic GPs. The temporal frequency of the stimuli was 20 Hz, with a single frame duration of ~0.05 s. In noise GPs, dipoles were randomly oriented. Instead, in noise RDKs, dots were placed in new random locations within the circular annulus on each successive frame [54]. Both visual stimuli had a variable coherence. To adapt the stimuli to each monitor size and resolution, we adopted the method of Li et al. [55] called the ‘card task’. It consisted of placing a credit card or a badge of the same size on the screen and adjusting a rectangle until it matched the dimensions of the card placed on the screen. The ratio between the rectangle’s dimensions (in pixels) and the real card is then calculated to obtain the logical pixel density (LPD). Based on the LPD, it is possible to present stimuli with set dimensions in pixels regardless of screen size.

### 2.3. Apparatus

The experiment has been programmed using the JavaScript library JsPsych [56] and carried out through the JATOS platform (JATOS version 3.5.8; Germany) [57]. All the observers took part remotely using their computers. The experimenters instructed the participants on the task via the Zoom platform (https://zoom.us (10 January 2022); USA).

### 2.4. Experimental Procedure

There were ten sessions in total: one pre-test, eight training sessions [41,47], and a post-test session. Each day, participants had to complete one session. The interval between each session was not longer than three days. Participants were instructed to conduct the sessions in a dark room. Participants sat at 57 cm from the screen. Participants were instructed to perform the experimental sessions at around the same time each day.

#### 2.4.1. Familiarization Procedure

**Figure 1:**
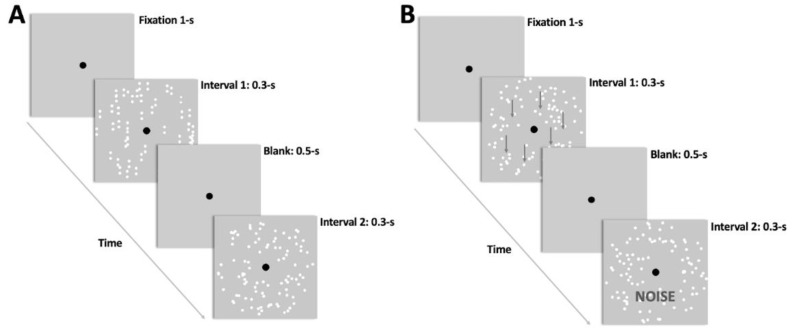
Schematic representation of the visual stimuli and the procedure used in the experiment. Two temporal intervals of 0.3-s each with the visual stimuli were presented after a 1-s fixation. (**A**) experimental procedure with Glass patterns (GPs), (**B**) experimental procedure with random dot kinematograms (RDKs). The first interval contains the coherent translational/vertical pattern and the second interval the random/noise pattern. However, in the experiment, this order has been randomized. In (**B**), the arrows are shown only for demonstrative purposes and were not presented during the experiment.

To familiarize themselves with the task, each participant completed a series of trials with both stimuli (i.e., dynamic GPs and RDKs). The coherent pattern was displayed in one interval, while the noise/random visual stimulus was delivered in the other. The participants had to report which temporal interval contained the coherent pattern by pressing the key ‘1′ on the keypad if the coherent pattern was contained in the first interval, and key ‘2′ if the coherent pattern was contained in the second interval. Each trial began with a 1-s black fixation point, followed by two 0.3-s intervals containing the visual stimuli and separated by a 0.2-s blank screen containing only the fixation point. The inter-trial interval was 2-s (see Figure 1).

#### 2.4.2. Pre- and Post-Test Assessments

The pre- and post-tests were divided into four blocks, each with 300 trials. The first two blocks were performed with dynamic GPs, while the last two blocks were performed with RDKs. Except for the post-test, which was preserved in the same blocks order as the pre-test, the blocks order has been counterbalanced. As part of the familiarization with the task, participants completed a two-interval forced-choice task (2IFC) (see Figure 1). A modified 1-up/3-down staircase [58] was used to estimate the 79% coherence threshold (see the Appendix A ). The coherence threshold was estimated by averaging the last 150 trials of the staircase. This phase lasted around ~1 h 15′.

#### 2.4.3. Training Procedure

The training sessions differed from the pre- and post-tests for the number of blocks and the visual stimulus presented. The group that was trained with RDKs performed eight training sessions with RDKs, whereas the group that was trained with GPs performed eight training sessions with dynamic GPs. In addition, each training session consisted of three blocks of 300 trials each. A single training session lasted ~45′.

#### 2.4.4. Data Analysis

The normality of residuals was assessed using the Shapiro–Wilk test. Data were analyzed using a Generalized Linear Mixed Model (GLMM) [59,60,61,62] with a ‘*lme4*’ package [62]. The analysis was performed in R (v4.1.3; Boston, MA, USA) [63,64]. A Gamma distribution and an identity link function were used in the GLMM model. We chose the Gamma distribution for the regression analysis because almost all the percentage discrimination thresholds fell into the Gamma quantiles, allowing for dealing with outliers without removing them [65] and because data were well approximated by a Gamma distribution. Data distribution was assessed with the function *fitdist* [66]. The link function in GLM(M)s determines the nature of the expected relationship between the predictors and the observed response. According to Lo and Andrews [67], a link function maps a nonlinear relationship to a linear relationship, allowing to fit a linear model to the data. A link function connects the transformed and original scales, allowing back-transformation to the original metric by providing a one-to-one mapping between the range of fitted values produced by the linear predictor on the transformed metric and the range of observed values on the original metric. If no transformation is required, and the observed response is assumed to tap the psychological construct to be measured, the function binding the expected values produced by the predictors to the dependent variable is the identity link function. In our case, an identity link function is more appropriate as we do not have strong reasons to apply a *log* or *inverse* transform to the dependent variable as the manipulation we introduced (i.e., training on either GPs or RDKs) is supposed to directly affect the coherence thresholds rather than some function of them. Additionally, since all the coherence thresholds estimated are above zero and thus away from the negative boundary, it is unlikely for the model to produce negative values. The normality of residuals was assessed using a Shapiro–Wilk test. Outliers were identified using the median absolute deviation with a cut-off of 3 [68].

## 3. Results

### 3.1. Analysis of Pre- and Post-Tests

**Figure 2:**
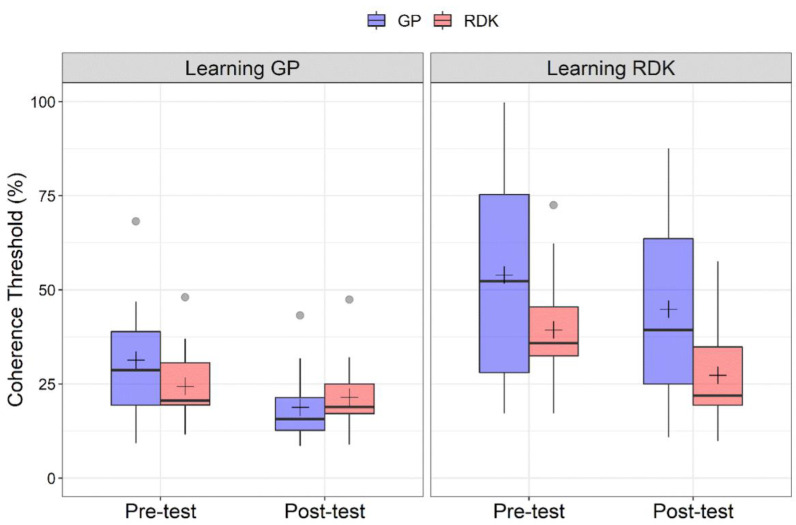
Boxplots of coherence thresholds (%) for the two types of training. (Left panel) Coherence thresholds for pre- and post-tests relative to the learning Glass pattern (GP) group, for GPs and random dot kinematograms (RDKs). (Right panel) Coherence thresholds for pre- and post-tests relative to the learning RDK group. For each boxplot, the horizontal black line indicates the median, whereas the black cross indicates the mean coherence threshold for that condition. Grey points indicate outliers. Data were plotted using R (v4.1.3; Boston, MA, USA).

Figure 2 shows the coherence thresholds estimated in pre- and post-training tests for each training group and stimulus type. The Shapiro–Wilk test showed that residuals for coherence thresholds were not normally distributed (*W* = 0.853, *p* < 0.001), with a high positive skewness of 1.45 (*SE*: 0.22). We also identified ten outliers that were included in the analysis. The GLMM included the learning group (learning GPs vs. learning RDKs), time (pre-training vs. post-training), and stimulus type (GP vs. RDK) and the interactions between all these terms as fixed effects. The subjects were the grouping variable. Fourteen models with different random effects (but the same fixed effects) were produced and compared. The best fitting model was selected using three different estimators of prediction error (*AIC*, *AICc* and *BIC*). The best fitting model from the three estimators of prediction error resulted in being a model with only random slope for time and stimulus type (no variation in intercept and correlation between random slopes for time and stimulus). The form of the best fitting model is as follows:

Coherence Threshold ~ Group * Time * Stimulus + (0 + Time + Stimulus|Subjects)

The regression analysis did report a significant effect of the group (*χ*^2^ = 11.627, *df* = 1, *p* < 0.001), time (*χ*^2^ = 20.680, *df* = 1, *p* < 0.001), stimulus (*χ*^2^ = 5.924, *df* = 1, *p* = 0.014), a significant interaction between group and stimulus (*χ*^2^ = 6.668, *df* = 1, *p* = 0.009), a significant interaction between time and stimulus (*χ*^2^ = 4.317, *df* = 1, *p* = 0.038), and a significant three-way interaction between group, time and stimulus type (*χ*^2^ = 8.313, *df* = 1, *p* = 0.004). The coefficients of the regression analysis are reported in Table 1, whereas the variance of random effects is reported in Table 2. It should be noted that the model explains most the variance, being the residual variance equal to 0.0487 (SD: 0.221). The conditional and marginal *R*^2^ were 0.999 and 0.519, respectively.

The learning GP group exhibited a lower coherence threshold than the learning RDK group (*M*: 23.09, *SE*: 2.41; *M*: 38.33, *SE*: 2.65 for learning GP and learning RDK, respectively), coherence thresholds in the pre-test assessment were higher than in the post-test (*M*: 35.02, *SE*: 2.29; *M*: 26.41, *SE*: 1.71 for pre- and post-tests, respectively) and translational dynamic GPs had overall higher coherence thresholds than RDKs (*M*: 34.35, *SE*: 2.64; *M*: 27.07, *SE*: 1.64 for GPs and RDKs, respectively).

For the group x stimulus interaction, Holm-corrected post hoc comparisons reported a significant difference between GPs for the learning GP and learning RDK groups (*p_adj_* = 0.004), and between RDKs always for the two groups (*p_adj_* = 0.0119), suggesting a difference in coherence thresholds between GPs and RDKs for the two groups, and a significant difference between GPs and RDKs in the learning RDK group (*p_adj_* = 0.003).

For the time x stimulus interaction, Holm-corrected post hoc comparisons reported a significant difference between pre- and post-tests for GPs (*p_adj_* < 0.001) and RDKs (*p_adj_* < 0.001), between GPs and RDKs for the pre-training assessment (*p_adj_* = 0.0139), and between GPs and RDKs for the post-training assessment (*p_adj_* = 0.0314).

For the three-way interaction, the most relevant Holm-corrected post hoc comparisons are reported in Table 3. In general, the three-way interaction reported a significant difference between pre- and post-tests for the GPs in the learning GP group, but not a significant difference between pre- and post-tests for RDKs (*p_adj_* > 0.05). The same applies to the learning RDK group in which we found only a significant difference between pre- and post-test for the RDKs, but not for GPs (*p_adj_* > 0.05).

**Figure 3:**
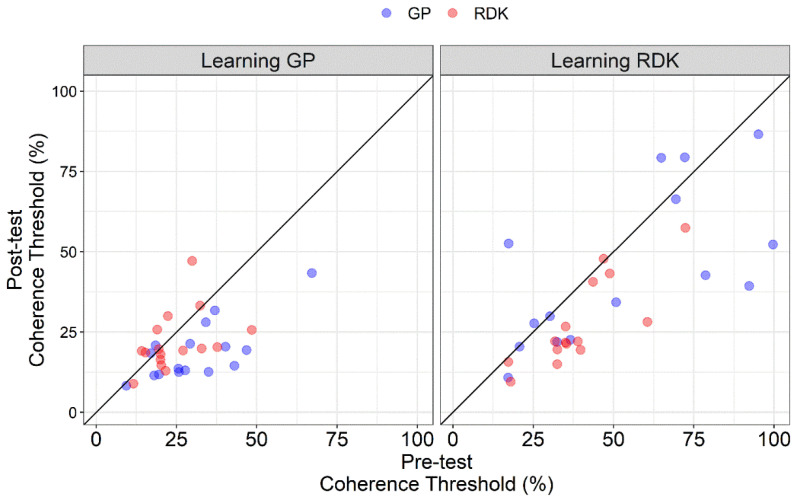
Scatter plot of the coherence thresholds (%) of individual participants in the pre-test and post-test assessments. Coherence thresholds are plotted separately for Glass patterns (GPs) and random dot kinematograms (RDKs) and learning group (i.e., learning GP and learning RDK). The diagonal line represents the same performance in the pre-test and post-test. All points falling under this line represent a better performance in the post-test than in the pre-test.

Figure 3 shows the coherence thresholds of individual participants in pre- and post-tests. The diagonal line represents the same performance in the pre- and post-test. All points falling under this line represent a better performance in the post-test compared to the pre-test. From the scatter plot, in the learning GP group, points relative to GPs fall under the equity line, suggesting an effect of VPL only for this class of stimuli when training participants with GPs. On the other hand, for the learning RDK group, most of the points relative to RDKs fall under the equity line suggesting an effect of VPL only for RDKs, while the points relative to GPs are more scattered. It is also clear how coherence thresholds in the learning RDK group are more widespread than in the learning GP group, further highlighting the difference in terms of sensitivity across the two groups and already shown in Figure 2. Taken together, these results suggest a high degree of specificity of VPL for translational dynamic GPs and RDKs.

### 3.2. Magnitude of Learning

Given differences in the initial and final assessment sessions between the two groups, and to compare performance across conditions, the magnitude of learning was calculated for trained and untrained stimuli as follows [69]:

(1) $$Magnitude\;of\;Learning=\frac{Post_{test}\;threshold-Pre_{test}\;threshold}{Pre_{test}\;threshold}$$

**Figure 4:**
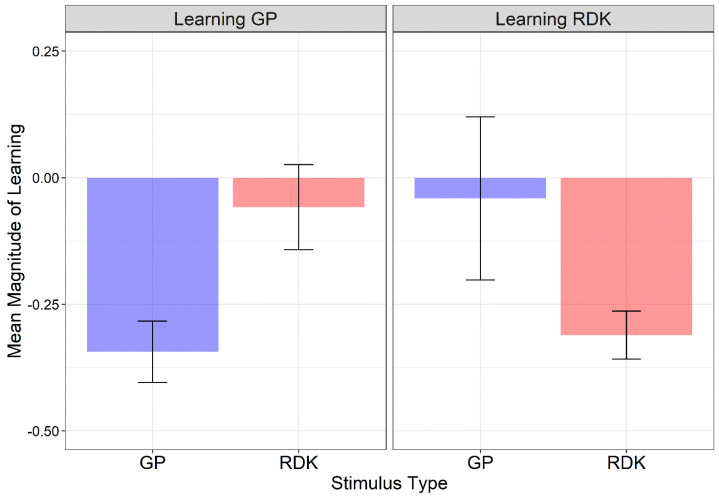
Mean magnitude index scores for each learning group and stimulus type. Larger negative values indicate greater learning. Error bars ± SEM.

This procedure allows for the assessment of any changes in performance based on the original performance level. Figure 4 shows the mean magnitude of learning for each learning group and stimulus type. The normality of residuals for each training group and each stimulus type was assessed with Q-Q plots and the Shapiro–Wilk test. Residuals were not normally distributed (*W* = 0.755, *p* < 0.0001) and two outliers were identified. Data were analyzed using the Align Rank Transform (ART), a procedure for the factorial non-parametric analysis of variance [70,71,72]. With this procedure, a linear mixed model can be implemented once the data are aligned and ranked for each main and interaction effect. Pairwise comparisons were conducted using the ART-C procedure [73]. A linear mixed model with random intercept across subjects and including the group (learning GP vs. learning RDK) as between-subjects factor and the stimulus type (GPs vs. RDKs) as within-subjects factor, revealed only a significant interaction between group and stimulus (*F*_1, 29_ = 16.63, *p* < 0.001) (group: *F*_1, 29_ = 0.023, *p* = 0.879; stimulus: *F*_1, 29_ = 1.061, *p* = 0.311). For the interaction between group and stimulus, Holm-corrected post hoc comparisons reported a significant difference only between GPs and RDKs in the learning GP group (*p_adj_* = 0.0089). For the learning RDK group, the high variability in coherence thresholds between the pre- and post-tests and the presence of an outlier (magnitude of learning = 2.03) might have biased the learning index by preventing a significant difference between GPs and RDKs for this group. In fact, uncorrected post hoc comparisons did report a significant difference between GPs and RDKs for the learning RDK group (*p* = 0.0431).

A series of one-sided one-sample permutation tests (sampling permutation distribution 5k) were performed on the magnitude index of each learning group and stimulus type to assess whether the magnitude of learning was lower than zero. A magnitude of learning equal to zero would indicate no learning for the trained stimulus and no transfer of learning to the untrained stimulus. The resultant *p*-values were corrected with the Holm method for two comparisons. The results showed that, for the learning GP group, the magnitude of learning index was significantly lower than zero only for GPs (i.e., the trained stimulus) (*p_adj_* < 0.0001), but not for the RDKs (*p_adj_* = 0.24). On the other hand, for the learning RDK group, the magnitude of learning index was significantly lower than zero only for RDKs (i.e., the trained stimulus) (*p_adj_* < 0.0001), but not for the GPs (*p_adj_* = 0.45). These results further suggest high specificity for the trained stimulus.

### 3.3. Learning Curves

**Figure 5:**
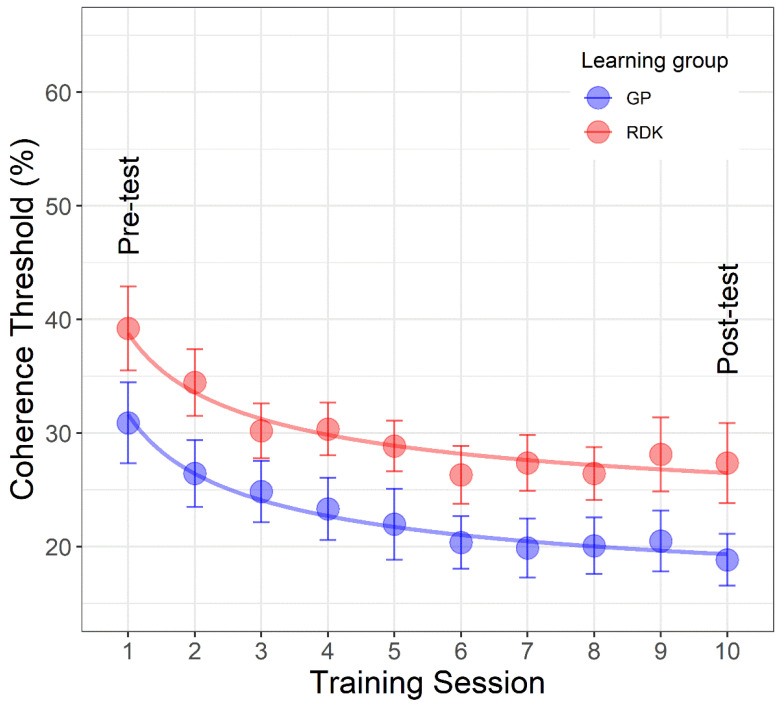
Mean coherence thresholds as a function of learning sessions separately for each learning group. The first point of each curve represents the pre-tests on the same stimulus used during the training, whereas the last point represents the post-test always on the same stimulus used during the training sessions. The dots in blue color represent the data for translational dynamic Glass patterns (GPs) and those in red for the random dot kinematograms (RDKs). The continuous lines represent the best fitting model (i.e., restricted model 4). Error bars ± SEM.

Figure 5 shows the mean coherence thresholds as a function of learning session. The first and last points of each curve represent the pre- and post-tests on the same stimulus used during the training. The learning curves were fitted with an extended power function [74,75,76] of the form:

(2) $$y=ax^{-b}+c$$

where *a* is the scale parameter, indicates the value of the power function in *x* = 1 (i.e., the pre-test assessment) and expresses the difference between the initial and the asymptotic performance (*c*), *b* is the learning rate (smaller values of *b* indicate slow improvements across learning sessions), *c* is the asymptotic coherence threshold after an arbitrarily large number of learning sessions, and *x* represents the amount of practice (i.e., learning sessions). The aim of this analysis was to assess for differences between the groups in terms of learning rate, whether the two groups differed from the outset (i.e., from the pre-tests assessments), and asymptotic performance.

We created a lattice of models from a fully saturated model to a maximally restricted model [74,75,76]. The fully saturated model consisted of six parameters (one *a*, *b*, and *c* parameter per learning group). The maximally restricted model, i.e., the model that postulated no change between learning groups, had only three parameters (*a*, *b*, and *c*), and assumed that the three parameters were the same across the two learning groups (i.e., no differences across learning groups). Between the fully saturated model and the maximally restricted model, a lattice of models having a different number of parameters was fitted (Table 4). The best-fitting model selection was based on the *F-test* (using the function ‘*anova*’ in R) to compare the models and assess which one provides the best parsimonious fit of the data. In the *F-test*, if the resulting *p-value* is higher than the significance level (0.05), then the simpler model is likely to be the preferred one. On the other hand, if the *p-value* is lower than the significance level, then the more complex model (i.e., the model with more parameters) is likely to be the preferred one. It should also be noted that the *F-test* can be used only with nested models (i.e., model A is nested in model B if parameters in model A are a subset of the parameters in model B). Comparisons between models with the same number of parameters (i.e., the same degrees of freedom) were performed using three estimators of prediction error (*AIC*, *AICc*, and *BIC*). In this case, the model with lower *AIC*, *AICc*, and *BIC* is likely to be the preferred model. Table 4 reports the lattice of models fitted to the coherence thresholds. All the possible pairs of models were compared without repetitions.

We found that the restricted model 4, i.e., the model consisting of the same parameters *a* and *b* across the two learning groups, but different asymptotic performance (parameter *c*) was the best fitting model. For the restricted model 4, parameters were: *a* = 18.34 (*SE*: 3.36), *b* = 0.482 (*SE*: 0.159), *c*1 = 13.298 (*SE*: 3.56), *c*2 = 20.45 (*SE*: 3.56) (*quasi-R*^2^ = 0.972). Restricted model 4 is the preferred model as it fits better than the fully saturated model (*p* = 0.339), than the maximally restricted model (*p* < 0.0001), and it has lower *AIC*, *AICc*, and *BIC* than the restricted models 5 and 6, having the same number of parameters. It also fits better than models with more parameters (i.e., restricted models 1, 2, and 3).

These results suggest that learning curves can be described by an extended power function that has the same starting coherence threshold and learning rate for the two groups, but a different asymptotic performance. The best-fitting model is reported in Figure 5.

## 4. Discussion

Using a VPL paradigm, we investigated the mechanisms underlying directional motion and form–motion integration in translational RDKs and dynamic translational GPs. The aim was to assess whether VPL could be transferred to the non-trained visual stimulus. Before and after the training sessions, the coherence thresholds for dynamic translational GPs and translational RDKs were measured in both groups (learning GPs and learning RDKs). After the initial assessment, participants were given eight training sessions [36,42,77]. VPL was found to be highly specific for the trained stimulus; that is, lower coherence thresholds in the post-test than in the pre-test were found for GPs and RDKs only when training with GPs and RDKs, respectively. Because training based on moving visual stimuli is direction selective [45], we found a lack of VPL transfer between GPs and RDKs probably because dynamic GPs do not elicit the percept of directional motion. To achieve learning effects, the motion direction of the visual stimuli employed must be suprathreshold and constant across trials [45]. While these characteristics may be guaranteed for RDKs, they are not met for dynamic GPs. As a result, we may state that, in dynamic translational GPs, VPL is dependent on global form characteristics than on apparent motion directionality. Moreover, training participants on a certain visual characteristic increases their sensitivity to the trained visual feature [45]. Thus, an increased performance correlates with a greater capacity to detect subtle visual variations. Consequently, after VPL participants could have become more sensitive to different motion directions, in the case of RDKs, and to different orientations in the case of dynamic GPs.

These results suggest that separate neural circuits control the processing of motion signals in translational RDKs and the processing of form–motion interaction in dynamic translational GPs. One possibility is that dynamic translational GPs are primarily led by the perception of global form, which necessitates the integration of local characteristics across space and time [78]. Swettenham et al. [78] using magnetoencephalography, investigated the perception of three different configurations of GPs: horizontal, circular, and radial. The authors found that the cortical visual area V3a plays a crucial role in the perception of global form in these three configurations. Precisely, the extrastriate area V3a is located between the primary visual areas (V1/V2) and higher-order areas; therefore, it represents an intermediate stage of visual processing. In a brain stimulation study, Pavan et al. [14] used rTMS to assess the causal role of early visual areas V1/V2 and hMT+ in the processing of static and dynamic translational GPs. Participants’ tasks were to discriminate which of two temporal intervals contained the coherent pattern, whereas the other interval contained a noise pattern (2IFC). The authors found that V1/V2 has a causal role in both static and dynamic translational GPs, whilst hMT+ has a causal role only in dynamic translational GPs. Based on these previous studies, the extraction of global form in translational GPs mainly relies on the activity of early/intermediate visual areas.

In terms of directional motion, evidence suggests that RDKs primarily engage higher-order extrastriate visual areas specialized in motion processing, such as the hMT+ [79,80,81,82,83,84,85,86]. Monkeys’ studies revealed that more than 90% of brain cells are responsive to motion in MT [81]. The posterior parietal regions are critical in the perception of moving stimuli, according to other physiological findings in macaque monkeys [87]. Neurons in the lateral intraparietal region (LIP) play an important role in motion direction discrimination [88]. In humans, LIP is analogous to a brain region near the intraparietal sulcus (IPS) [89]. Accordingly, in healthy participants, the transient inhibition of hMT+ by mean of rTMS can impair for a few milliseconds the perception of motion direction in circular RDKs [90]. Interestingly, Sterzer et al. [91] investigating the perception of RDKs showed that also the activity of the early level of the visual system (i.e., V1) is mediated by feedback from hMT+. In conclusion, whereas the processing of translational RDKs and dynamic GPs might rely on overlapping cortical visual regions, the role and importance of these areas may differ.

Despite the present study suggests that the perception of dynamic translational GPs and RDKs relies on different processing mechanisms, when training with RDKs, there is a small amount of transfer, although not significant, for untrained GPs (Figure 2). In this case, we can speculate that the influence between form and motion is slightly asymmetric: from motion to form but not from form to motion. Studies on biological motion demonstrated how motion is important to animate form features contained in an image [92]. However, other authors found an opposite asymmetry; from form to motion [8,93]. In particular, Or et al. [93] showed that global form impacted the perception of global motion direction more effectively than how global motion affects the perception of global form. Differently, the global orientation of the stimulus was not influenced by motion direction. The interplay between form and motion cues in dynamic GPs and RDKs needs to be further investigated and more evidence is needed to shed light on the lack of learning transfer. 

In this context, various research has found that task difficulty influences transfer [39,42,94,95]. For example, Liu and Weinshall [95] performed a study on the perception of motion direction based on VPL to investigate learning transfer effects. Participants had to discriminate eight different motion directions. The authors found almost no perceptual transfer when directions had a large difference, but a learning transfer occurred for similar motion directions. Finally, they found VPL transfers in easy but not difficult tasks. In our case, conditions and task difficulty have remained homogeneous amongst the groups. Therefore, the lack of perceptual transfer, especially in the learning GP group, is unlikely to depend solely on task difficulty. Additionally, in each training block and session, the staircase always started from the maximum coherence level, thus making the task easier [39,96].

Moreover, we observed lower coherence thresholds for RDKs than dynamic GPs in both learning groups. Participants perceived translational RDKs easier than dynamic translational GPs [23]. This probably occurred because the human visual system is engaged in a twofold task in dynamic GPs: global form processing and motion processing. Additionally, we found a high inter-observer variability between the two learning groups. The learning GP group showed lower coherence thresholds in all training sessions compared to the learning RDK group. A similar inter-group variability has been found in other VPL studies [41,69,97]. Differences in the initial level of performance and VPL rate are common in experimental paradigms with VPL, even in homogeneous groups of participants where age, education, and motivation were controlled [97]. To control for groups’ inter-individual differences, we evaluated the magnitude of learning (Figure 4). We tested the changes in participants’ coherence thresholds considering the initial performance of each individual observer [69]. We found a significant difference in the magnitude of learning between the trained and the untrained visual stimulus only for the learning GP group. Moreover, we found that the magnitude of learning was not significantly different from zero for the untrained stimulus in both learning groups, suggesting that VPL is highly specific for the stimulus trained.

In conclusion, though there is brain imaging evidence that both dynamic GPs and RDKs activate some overlapping cortical areas (e.g., hMT+, V3) [21,22], the neural circuits underlying the processing of the two stimuli appear to be somehow different. Dynamic translational GPs could be mainly processed and perceived for their form signals [23], and VPL is likely to occur at early and intermediate levels of the visual system. On the other hand, translational RDKs are processed and perceived for the motion signals, and VPL is likely to take place at higher levels along with the visual system. Future studies may explore whether complex configurations such as dynamic circular GPs and circular RDKs show the same learning specificity or exhibit a certain degree of transfer.

## Figures and Tables

**Table 1:** Estimated coefficients of the best fitting generalized linear mixed model (GLMM). Standard Error (*SE*), *t*- and *p*-values for predictors (including intercept) are listed.

Predictors	Estimate	*SE*	*t*-Value	Pr (>|*t*|)
(*Intercept*)	28.01	2.953	9.485	<0.0001
Learning Group	19.746	6.341	3.114	0.0018
Time (pre/post)	−10.697	2.677	−3.996	<0.0001
Stimulus (GP/RDK)	−5.430	3.645	−1.490	0.1362
Group * Time	2.475	4.478	0.552	0.580
Group * Stimulus	−5.399	5.742	−0.940	0.347
Time * Stimulus	7.819	2.350	3.328	0.0008
Group * Time * Stimulus	−12.220	4.238	−2.883	0.0039

**Table 2:** Variance of the random effects.

Name	Variance	SD
Time (Pre-test)	244.1305	15.6247
Time (Post-test)	155.3663	12.4646
Stimulus (RDK)	152.6115	12.3536
Residual	0.0487	0.2208

**Table 3:** Selected post hoc comparisons for the three-way interaction between group, time, and stimulus type. *p*-values are adjusted with the Holm method for 28 comparisons.

Group	Time	Stimulus	*p*-Value
Learning GP Learning RDK	Pre-test	GP	0.0277
Learning GP	Pre-test Post-test	GP	0.0013
Learning GP Learning RDK	Post-test	GP	0.0003
Learning RDK	Post-test	GP RDK	0.0031
Learning RDK	Pre-test Post-test	RDK	0.0015

**Table 4:** Lattice of power law functions. The fully saturated model has six parameters, whereas the maximally restricted model has three parameters. *f*1(*x*) indicates the function fitted to the learning Glass pattern (GP) group, and *f*2(*x*) indicates the function fitted to the learning random dot kinematogram (RDK) group.

Function Name	Equation	Number of Parameters
Fully Saturated	$f1\left(x\right)=a1x^{-b1}+c1$ $f2\left(x\right)=a2x^{-b2}+c2$	6
Restricted 1	$f1\left(x\right)=ax^{-b1}+c1$ $f2\left(x\right)=ax^{-b2}+c2$	5
Restricted 2	$f1\left(x\right)=a1x^{-b}+c1$ $f2\left(x\right)=a2x^{-b}+c2$	5
Restricted 3	$f1\left(x\right)=a1x^{-b1}+c$ $f2\left(x\right)=a2x^{-b2}+c$	5
Restricted 4	$f1\left(x\right)=ax^{-b}+c1$ $f2\left(x\right)=ax^{-b}+c2$	4
Restricted 5	$f1\left(x\right)=ax^{-b1}+c$ $f2\left(x\right)=ax^{-b2}+c$	4
Restricted 6	$f1\left(x\right)=a1x^{-b}+c$ $f2\left(x\right)=a2x^{-b}+c$	4
Maximally Restricted	$f\left(x\right)=a^{-bx}+c$	3

## Data Availability

The data presented in this study are openly available in Open Science Framework (OSF) at DOI 10.17605/OSF.IO/K7UCE.

## Appendix A. Modified 1-Up/3-Down Staircase

We define coherence as the number of dots (in the case of RDKs) or dipoles (GPs) that drifted in the same direction or had the same orientation. The coherence ratio is the ratio between the coherence and the total number of dots or dipoles of the trial. The initial trial coherence was equal to the total number of dots/dipoles, therefore with a coherence ratio of 100%. We define accuracy as the ratio between the number of correct answers over the number of total answers to a set of trials. We decided to let the coherence decrease if the participant answered three times in a row correctly, while only one wrong answer was enough to let the coherence increase. We call reversal the set of answers given between two changes in the coherence slope. For example, if a participant:

- gave 6 good answers in a row (2 sets of 3 correct answers);
- then gave 2 wrong answers;
- then gave 2 good answers;
- then gave 1 wrong answer;
- then gave 3 good answers.

The coherence was supposed to decrement twice, then increment three times, then decrement once. The answers from b) to d) included are part of the same reversal. Moreover, the first 10 staircase steps increased (or decreased) the coherence by 100, 65, 42, 27, 18, 11, 7, 5, 3, 2 dots (or dipoles) at a time—then by 2.

We let the coherence increment when the accuracy of the last 16 reversals’ answers was below a threshold value of 0.79 and decrease only when above this threshold. This value was taken as the asymptotic convergence of a Levitt 1-up/3-down staircase (from which this method took inspiration). We were able to let the experiment have a faster convergence, to let the tester be around its coherence threshold for more time and thus collect a more reliable value. The staircase was terminated after 300 trials and the coherence threshold was calculated by averaging the coherence estimated in the last 150 trials.
